# Fever pitch: Coloniality and contention within community health’s yellow fever response in Kenya

**DOI:** 10.1080/17441692.2025.2519659

**Published:** 2025-06-22

**Authors:** Kathy Dodworth, Brenda N. Mukungu

**Affiliations:** a Centre of African Studies, University of Edinburgh, Edinburgh, UK; b Department of Anthropology, Gender and African Studies, University of Nairobi, Nairobi, Kenya; c Independent Scholar, Nairobi, Kenya

**Keywords:** Yellow fever, vaccinations, Kenya, community health, coloniality, SDG 3: Good health and well-being, SDG 6: Clean water and sanitation, SDG 10: Reduced inequalities, SDG 16: Peace, justice and strong institutions

## Abstract

In January 2022, a number of Yellow Fever cases were identified in Kenya’s Isiolo County for the first time, triggering a national-level response centred on vaccinating residents. 181,000 people were vaccinated in July, around 72% of the eligible population. In the face of this ostensible success, this article explores the continuing coloniality, that is, long-standing patterns of domination, operating within disease control in Kenya’s northeast, whereby punitive encounters with the state loom large. Despite health matters being devolved, top-down implementation from nationally-controlled actors exacerbated local distrust, resulting in contention around the roll-out and of the authorities behind it. This article, drawing on ethnography supplemented by in-depth interviews and Focus Group Discussions over 12 months 2022-2023, centres the experiences of Community Health Volunteers (CHVs) over the ten-day campaign. We adopt a Fanonian lens to interpret our findings, historicizing the contention CHVs faced from their communities, in a region where governmental approaches oscillate between neglect and heavy-handed remedial action. We operationalise Fanon’s ‘psychoexistential complex’, whereby CHVs internalise the conflict between their roles of community representative and state-enforcer, exacerbated by their precarity and invisibility to others. We conclude with a call for CHVs’ place to be protected, capacitated and seen within outbreak response.

## Introduction

Infectious disease control has a contentious history in Africa. The containment of infectious disease was the cornerstone of ‘tropical medicine’, leading to some of the most coercive, top-down practices in colonial governance (Packard, 2016). The earliest international agreements focused on the containment of disease by European colonizing powers from the 1800s, strengthened over subsequent decades (Sirleaf, 2023), driven primarily by traditional and emerging powers’ geopolitical concerns (Sealey, 2011). More broadly, Western medical discourse on Africa helped craft the alterity of the continent as ‘a repository of death, disease and degeneration’ (Vaughan, 1992, p. 14), legitimating intervention by colonial powers over the long durée. Othering and alterity continue to infuse public health management today, whereby epidemics represent the ‘dark side of modernisation’ and ‘impossibility of securing the body politic’ in today’s world (Kelly et al., 2019, p. 1). These dynamics, we contend, are heightened when epidemics break out in marginalised, indeed maligned, regions.

Isiolo County, the focus of this article, is one such ‘threat region’: historical gateway to the ‘ungovernable’ northeast of Kenya (Amutabi, 2005; Kochore, 2020), still framed as threat today (Shanahan, 2016). In January 2022, a number of Yellow Fever (YF) cases were identified in Isiolo for the first time, and, in early March, national government declared the situation an outbreak. In July, it implemented a national-level incident response programme to vaccinate Isiolo’s population. 181,000 people were vaccinated out of an eligible population of approximately 250,000, achieving a vaccination rate of 72% and the outbreak abated. Given Isiolo’s historical containment, whereby movement was curtailed by force and the northeast sequestered from settler Kenya (Hogg, 1980; Khalif & Oba, 2013; Whittaker, 2017), we benefit from the recent revival of Fanonian insights into epidemic response in Africa (S. H. Ali & Rose, 2022; Farmer, 2021; Hirsch, 2020) to understand local contention in the YF response. Focussing on community health, we contribute to this scholarship in three ways. First, we contextualise the response, whereby Isiolo's history of sporadic and violent contact with the state is pertinent to understanding contemporary dynamics. Distrust of but also denigration by external players fuelled rumour and anger on the part of health volunteers, leading agitation to reach ‘fever pitch’: a heightened state of emotion and angst. Second, we observed a ‘spectrum of contention’ on the part of community members, who contested not necessarily whether the disease was *real* but rather its forceful management.

Last, we draw on Fanon’s anti-colonial insights to probe the multiple, contradictory positionalities of Community Health Workers (CHWs), known in Kenya at the time of research as Community Health Volunteers (CHVs). CHVs are trusted, locally-embedded volunteers on the one hand, yet enforcer of national interventions on the other (Kok et al., 2017; Nading, 2013). CHVs use their embeddedness to overcome local ‘resistance’ or ‘hesitancy’, which is a core part of Kenyan health policy (Osur et al., 2022), yet find themselves complicit with coercion and subject to contention. Further, CHVs expressed feelings of dehumanisation when demeaned or displaced by professionals and outsiders, heightened in this emergency setting. We draw on Fanon’s ‘psychoexistential complex’ (1952, p. 14) at an individual level, linking in the discussion to his statements on the pitfalls of nationalisms (1967) that continues to denigrate those on the margins.

### Fanon and the coloniality of being

We centre Fanon’s work for a number of reasons. The first, more empirical, is that Fanon explicated how public health interventions were at the forefront of colonial rule (1965). The doctor or, relevant to our case, teacher (1967, p. 60) were defining points of contact with a distant state, enacting forms of discipline and compulsion. The second is onto-epistemological, whereby Fanon explored the impact of colonialism (which we broaden to ‘coloniality’) on two levels. One level is individual: the ‘psychoexistential complex’ central to *Black Skin, White Masks* (1952) borne of the everyday sequestering and differentiation of the colonised. While Fanon believed the juxtaposition between Black and White races engendered collective inferiority, we explore how ‘new oppressors’ in Kenya perpetuated colonial-like subjection following independence (Tabensky, 2010). The other is structural, whereby Fanon examines the violence that enacts differentiation. Violence is structural where societal factors prevent certain groups from self-realisation and parity (Galtung, 1969). For Fanon, a violent ‘imaginary’ differentiates those on the margins (Mbembe, 2001, p. 175), allowing neglect to take place. Fanon forewarned in the *Wretched of the Earth* (1967) of new exclusions and violence within post-independence ‘state nationalisms’, which were viscerally realised in Kenya’s ethnicised politics (Murunga, 2004; Nasong’o, 2015).

We believe a Fanonian lens enables multi-scalar work that centres the embodied psychosocial effects of colonialism, while attending to the structural violence that enacts such. In Kenya, the colonial inheritance allowed for renewed differentiation and dispossession, based on ethno-linguistic and gendered hierarchies, leaving behind Fanon’s monolith ‘blackness’. Thus, post-independence, we employ the concept of coloniality (Mignolo, 2005; Quijano, 2007), which refers to ‘long-standing patterns of power that emerged as a result of colonialism’ (Maldonado-Torres, 2007, p. 243) that evolve within new ethno-political landscapes. Maldanado-Torres’ ‘coloniality of being’ (2007) spotlights all ‘dehumanizing lines of differentiation’ in the postcolonial present. The neglect of and inequity in Kenya’s north is sustained by a violent imaginary: that is, the differentiation between groups as everyday ‘cultural praxis’ (Mbembe, 2001, p. 175).

### Containment and coloniality in Kenya***’***s North

From the mid-colonial era, Kenya’s north was governed by ‘containment rather than engagement’ (Odhiambo, 2014, p. 7). Isiolo County was originally part of the Northern Frontier District, established by the British at the end of the nineteenth century (Whittaker, 2017). With semi-arid pastoralist areas deemed of little economic interest, Isiolo Town was militarised in the 1900s as buffer to threats from the north, including disease (Amutabi, 2005; Kochore, 2020). Northern inhabitants were deemed ‘unruly’, who neither ‘required’ nor ‘merited’ development (Whittaker, 2017, p. 386, citing colonial archives), with infrastructure neglected (Wario Arero, 2007). Nomadic movement was curtailed through ordinances from the 1900s, by force from the 1930s (Khadiagala, 2010), whereby residents were subject to arrest, fines or ‘collective punishments’ on entire villages (Odhiambo, 2014; Whittaker, 2015). Movement restrictions caused the degradation of communal resources, exacerbating drought and conflict (Wagura, 2016). The ‘defining feature’ of contact with the state was punishment (Whittaker, 2015, p. 645), mapping onto Fanon’s spatial power and compartmentalisation between settler and native (1967, p. 30).

Independence represented continuity over change, with the northeast demarcated as ‘low potential’ by the new government (Wagura, 2016, p. 295). Immediately after independence in 1963, Somali-speaking insurgents in the district, sponsored by the Somali Republic, fought for secession until around 1969 in what was known as the Shifta Wars (Khalif & Oba, 2013). The government’s response was severe, declaring a state of emergency wherein security forces enforced a raft of measures, including curfews, detentions without warrant and forcing pastoralists to settle in ‘security villages’ (Khalif & Oba, 2013; Whittaker, 2017), shooting those who strayed (Hogg, 1980). Collective punishment continued until the 1990s (Whittaker, 2015, p. 649). These actions led to huge losses of life and livestock: levels have not recovered, producing intergenerational harms (Khalif & Oba, 2013; Pike et al., 2010). Given such, independence held little significance for pastoralists, who ‘did not consider themselves citizens’ (Moskowitz, 2019, p. 130; Wario Arero, 2007) but rather signified the ‘time of stop’, whereby the autonomous pastoralist life ended (Khalif & Oba, 2013). This divide remains palpable in remote regions (Kochore, 2020), with interlocutors teasing us: ‘you are not in Kenya now!’, meaning Kenyan laws did not apply. One CHV was nicknamed ‘Kenya’ by those she served: ‘the language I speak is from Kenya, not here. It's like they don’t consider this place part of Kenya’ (Interview).

Containment was discernible within colonial health, devised to shield settlers, improve labour forces or proselytise, with post-independence priorities remaining focused on urban centres (Chaiken, 1998; Mburu, 1981). Kenya’s pastoralist, conflict-affected and increasingly Muslim northeast fell outside most national development drives, with the exception of irrigation and water (given the growing importance of its animal produce). From the 1980s, President Moi initiated decentralisation reforms, although funding became diverted to his political heartlands (Barkan & Chege, 1989). Irrigation schemes were initiated in Isiolo but remained incomplete or mismanaged, leading to new areas of stagnant water and favourable conditions for zoonotic diseases (Dzingirai et al., 2017). Despite expanding investment in health from the 2000s (Ministry of Health, 2014) and the positive impact of decentralisation following constitutional reform (Masaba et al., 2020), northern pastoralists lagged ‘considerably behind’ other regions with regard to services, safe water and in most development indices (Noor et al., 2009; Wagura, 2016). Adult literacy in the northeast was by far the lowest at 9%, compared to the 62% average (Government of Kenya, 2006), and remains depressed today (Isiolo County, 2023). Yet, successive policymaking has placed pastoralism at the root of its own underdevelopment (Odhiambo, 2013). Media coverage of pastoralist areas remains overwhelmingly negative, newsworthy only as a cause of ‘conflict and disease’, even though journalists themselves recognise such biases (Shanahan, 2016, p. 408).

Today, Isiolo County remains expansive and diverse. While low-populated, its population almost doubled between 2009 and 2019, with almost half residing in Isiolo Town in the southwest of the elongated county (Kenya National Bureau of Statistics, 2019). It has five ethno-linguistic groups in significant numbers: the pastoralist Borana, Somali, Samburu and Turkana, and the sedentary Meru (Boye & Kaarhus, 2011; Dibondo, 2019), predominating in different areas. Almost 70% of Isiolo is Muslim, primarily the Borana and Somali populations, as compared to Kenya’s 85% Christian population as a whole (Kenya National Bureau of Statistics, 2019). These dynamics sit against a backdrop of visible environmental breakdown that threatens pastoralist livelihoods, on which 80% of the county depend (Isiolo County Government, 2023). Isiolo thus houses groups historically excluded from state-making (Moskowitz, 2019), which is why the community health model proved so impactful to Isiolo’s healthcare (Dodworth & Mukungu, 2023), as well as why non-state actors had driven development more broadly (Brass, 2016; Dodworth, 2022; Hearn, 1998). However, the county government had assumed overall control by the early 2010s. At the time of research, there were formally ∼760 CHVs across ~56 health units, serving an estimated population of 289,000 (Isiolo County Government, 2023).

We contend that Isiolo's violent history with an unreliable, punitive and remedial state remains pertinent to understanding the dynamics of the YF campaign in 2022.

### Yellow fever in Kenya

The first recorded outbreak of Yellow Fever (YF) in Kenya, an acute haemorrhagic illness transmitted by infected mosquitoes, occurred in 1992 in Rift Valley Region, with subsequent outbreaks in 1993, 1995 and 2011 (Uwishema et al., 2022). These outbreaks were likely sustained by sylvatic (forest) vectors, which require specific, short-lived environmental conditions (Ellis & Barrett, 2008). However, YF has three transmission cycles: sylvatic, savannah and urban (Gianchecchi et al., 2022). In the sylvatic, monkeys that live in rainforest are infected through mosquito bites and the virus transmitted to humans via mosquitoes. In savannah, transmission takes place both from monkeys to humans and between humans, via infected mosquito bites. The urban cycle involves transmission from mosquitoes to humans, brought to urban areas by infected humans. While outbreaks are normally triggered by the first and second cycles, in Isiolo, it was the third (Uwishema et al., 2022).

**Figure 1:**
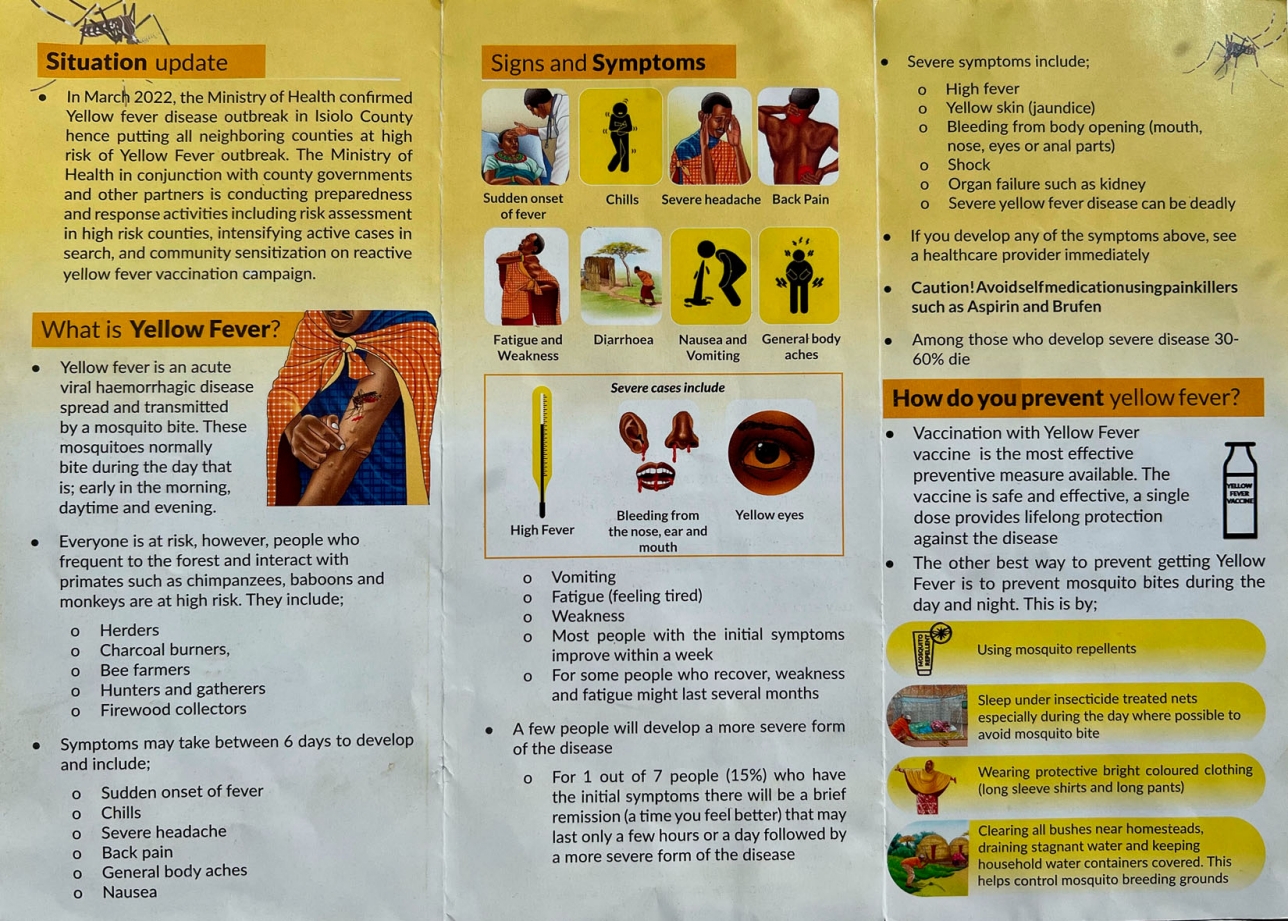
Repurposed yellow fever information leaflet, circulated predominantly by CHVs.

Given that common YF vectors are less prevalent in semi-arid areas like Isiolo, the 2022 outbreak was unexpected. Information leaflets (Figure 1) focused on other cycles, warning against proximity to primates yet, in Isiolo, YF was maintained via primary vectors, specifically mosquitoes breeding in water stored around homes and areas of stagnant water. The growth of urban areas with limited sanitation, the porous nature of nearby borders and the movement of people from rural to urban areas were considered predisposing factors (Uwishema et al., 2022). Lastly, YF transmission was exacerbated by low population immunity (WHO, 2022), feeding fears that YF could move rapidly to urban centres. Kenya has a low vaccination rate as compared to other at-risk countries: an estimated 18.5% of under-five children are vaccinated, with lower rates in urban areas (M. S. Ali & Mekonen, 2024). YF is not included in Kenya’s routine vaccination schedule for children, with the exception of four at-risk counties around the northern Rift Valley (Ministry of Health, 2020, p. 48), where rates were marginally higher (M. S. Ali & Mekonen, 2024, p. 4). In comparison, in Ghana, where there have been similar outbreaks, vaccination is routine and rates around 88% (M. S. Ali & Mekonen, 2024, p. 4).

The Isiolo outbreak was declared an emergency by Kenya’s MoH following a WHO declaration in early March 2022. Cases totalled 53, of which two thirds occurred in the remote Merti sub-county (WHO, 2022). National government implemented top-down measures via its National Incident Management Structure, with surveillance and rapid response teams deployed to assess at-risk populations and integrate vector control measures (WHO, 2022). MoH and WHO co-financed the response, the latter fulfilling a request for vaccines to be utilised across Isiolo, plus adjacent counties considered high risk (Uwishema et al., 2022). The drive targeted those aged nine months to 60 years, using schools and health facilities as well as mobile administration, declaring 72% vaccinated by the end of the campaign. According to Isiolo’s Emergency Plan of Action, MoH was responsible for the vaccination campaign and county government for the emergency response (IFRC, 2022, p. 4), although roles and responsibilities blurred between levels and actors, including within community health. Schools, religious leaders and chiefs were also used to inform and mobilise residents. Messaging focused on radio in town and loudspeakers in rural areas, on top of the leaflets circulated predominantly by CHVs.

Media reporting emphasised YF’s deadliness and need for containment. In a story titled *‘Kenya declares yellow fever outbreak after three die in Isiolo*’ on 5 March, The East African quoted the Director General for Health on the need for national alarm, citing risk from remote northern counties (Oketch, 2022). The story was picked up by foreign press after a fourth death on 8 March, emphasising YF’s virulence within a remote pastoralist region (Agence France-Presse, 2022). In May, a report in The Star titled ‘*Yellow Fever continues to spread*’ highlighted the lack of mass vaccination, leading to Kenya’s unwanted high-risk status with the WHO (Muchangi, 2022). In isolation, this was all accurate, given the seriousness of YF in areas of low immunity. Placed in historical context, such reporting affirmed biases whereby pastoralist areas are only newsworthy as a reservoir of ‘conflict and disease’ (Shanahan, 2016, p. 408), taken to extremes in Kenya (Shanahan, 2016, p. 411). Given that arid and semi-arid areas cover 80% of Kenya’s landmass (Odhiambo, 2014), this bias highlights the dense concentration of power in Kenya and how an ‘imaginary’ of its backward hinterlands legitimates cycles of neglect and remedial action.

While the outbreak response was of course shaped by inputs from powerful global bodies like the WHO, its contention was thus determined by the coloniality *within* Kenya’s post-independence settlement. Beyond infrastructural gaps and mismanagement, which were predisposing factors for YF, the response was mediated by suspicion as to why this drive was prioritised at a time of broader crisis. For CHVs, who ‘bridge’ national priorities with those of their communities, this led to disjuncture. CHVs are governed by healthcare policies, at the time the Community Health Policy 2020–2030 (Ministry of Health, 2020) under Kenya’s broader health policy 2014–2030 (Ministry of Health, 2014) whereby community health comprises ‘Tier 1’ of the health system. However, CHVs’ roles in emergency response are unclearly specified. Community Health as a whole, which includes salaried staff such as Community Health Assistants (CHAs), are expected to ‘conduct disease surveillance’ and ‘promote immunisation’ (Ministry of Health, 2020, p. 21). In practice, there are marked differences in CHAs and CHVs with regards to the kinds of work that they do, their security and positionality, which, as we explore, compounded the angst of CHVs.

## Methodology

The data presented centre on observations captured in fieldnotes over the ten-day YF campaign 21–31 July 2022, supplemented by 80 in-depth interviews conducted for a larger qualitative study. Data collection for the full study took place in Isiolo County March 2022 – Mar 2023, focusing on the life stories of CHVs. Interviews were mainly unstructured, designed to allow CHVs to tell their stories as they saw fit. Interviews took place in Swahili interspersed with English terms, mainly in CHVs’ homes. We conducted 61 days of ‘negotiated interactive observation’ (Wind, 2008) of CHVs at work, including mobilisations, between household visits, vaccination drives and meetings. We adopt Wind’s framework given that we made no claim to be full participants and our presence in CHVs’ working lives, rather than hinging on one moment of consent, required ongoing dialogue. We conducted six Focus Group Discussions (FGDs) on CHVs’ experiences of digitisation, which concluded with a discussion on ‘empowerment’. We recruited CHVs through snowballing and later observing monthly meetings, identifying the more vocal as well as the quietly disengaged across the county. CHVs signed informed consent forms for each activity and were compensated with a travel allowance. The study was approved by The University of Edinburgh’s School of Social and Political Science Ethics Board and by the Kenyan National Commission for Science Technology and Innovation in March 2022. Its instruments and protocols were reviewed in a workshop with Isiolo County staff and CHVs in May 2022, after which staff issued county permissions to collect data and observe activities.

Fanon eschewed psychological reductionism, putting him at odds with his contemporaries. Instead, he viewed the ‘subject as dynamic inter-relationality’, ‘part of a larger field of social and cultural arrangements’ (Maldonado-Torres, 2017, p. 434). We drew on situated, mobile ethnographies (Evans & Jones, 2011), shadowing four senior CHVs in particular. These four worked in teams that comprised vaccinators (mostly nurses), public health officers (PHOs) and monitors. We centred on three public vaccination centres (two schools and one health facility), where CHVs were tasked with scribing, mobilising and gaining consent, wherein the vaccination process, including our role writing certificates, was structured. However, vaccinations also occurred wherever vaccinators put down their insulated bags, whereby Mukungu’s role became one of background observer, although would still interact with community members. Being mobile enabled us to observe CHVs’ inter-relationality with communities and various authorities, all the while narrating their frustrations to us.

While observing brings an assumption of ‘outsiderness’, there is constant, if not strategic flux between insider / outsider (Aberese-Ako, 2017; Dodworth, 2021). We were not detached observers but embraced our situatedness (Haraway, 1988), drawing on pre-existing relationships with CHVs to gain access and information in a fast-changing environment. In some scenes, we slotted into a textbook participant observer role, writing hundreds of vaccination certificates in schools while grappling, as others, with local names and spellings. At other times, a lack of pre-existing relationships with county staff, external teams and community members generated suspicion. Dodworth, as a white European female, withdrew from certain activities. Mukungu, as a Black Kenyan female, was congruous to the scene to some, such as children and teachers, and incongruous to others, such as local health staff. Though our purpose was made clear at the start of each activity, we were seen at different times as evaluators, researchers, advocates and confidantes.

We conducted an inductive thematic analysis (Braun et al., 2018), coding interviews under themes using NVivo 14 software, while fieldnotes were coded manually. There are limits to the study. First, we did not interview community members, exploring community relations via CHV observations, interviews and FGDs. Second, we did not gather data on YF across all interviews, given these were unstructured and the heat of the campaign subsided. We have therefore drawn from data on COVID-19 and, to a lesser extent, Human papillomavirus (HPV), to explore adjacent imprints of top-down vaccination campaigns. While the origins, demographics and epidemiology of these diseases differ, CHVs linked experiences of successive campaigns, both in their minds and those they serve. Thus ethnographically, interventions regarding YF interwove with the ‘layered fragments of earlier epidemic events’ (Geissler & Prince, 2020, p. 253; also Lowes & Montero, 2021). Our findings are organised into exploring CHV displacement from above; contention by communities from below; and the psychoexistential angst that CHVs experience due to their denial.

## Results

### CHV displacement

On a blustery day in July 2022, we were scheduled to interview a senior CHV, who we will call Fatuma. On our way to her home dispensary, she called us to ask that we reroute to a larger facility. As we arrived, we saw senior CHVs gathered who were known to us:

A number of CHVs had congregated at the entrance of the main hall and I think they were awaiting communication as to what would transpire that day. Nobody had a clue about the procedure; there were speculations about a training for the CHVs and the vaccinators. (Fieldnotes, July 2022)^1^

Conjecture was a normal part of being on ‘permanent standby’ for community health work, but uncertainty and conflicting information remained a feature throughout the campaign. With suggestions that the exercise could take many days, this had potentially large repercussions, whether via income top-ups or reorganising responsibilities. Fatuma half-heartedly suggested we wait to see if we could interview her at that venue, should she be released. However, the uncertainty, manifest in the growing anxiety and frustration on the faces of CHVs, led us to abandon the plan. We ‘hung out’ for some hours on the side-lines, trying to make sense of what was coming, before the crowd dissipated without conclusion.

The following day, Mukungu returned, based on information from the clinical officer, to the same venue to learn that there was a training at the main hospital. She made her way there to find no sign of CHVs or vaccinators, so returned home to await further instructions. On the third day, senior CHV Yusuf informed Mukungu that he too had used his own means to try multiple venues on the basis of conflicting information, before finally attending a short briefing. In the end, most of the CHVs that had first assembled were not selected, missing out on ten days’ facilitation fee. The fact that many had paid for taxis to facilities on the assumption of reimbursement raised the temperature further.

These vignettes offer insight into ways that CHVs are routinely reminded of their place as dispensable volunteers. The issue of payment went beyond purely materialistic motivations as rather a means that CHVs might feel valued and *seen*. When CHVs found themselves excluded, it was painfully experienced in what Mbembe terms ‘the violence of being reduced to nothingness’ (2001, p. 144). Often in our time together, Yusuf linked the devaluing of CHVs’ time to their denigration as volunteers, extended to ‘conspiring’ against CHVs on the part of staff:

Yusuf echoed his sentiments on how they are always thought to be important on paper but treated with no value when it comes to work where some incentive is in question. He said that the selection and recruitment process was marred with nepotism and favouritism and was not transparent enough. He wondered why [salaried health assistants] were part of mobilization yet some CHVs were left out. ‘That is not fair!’ (Fieldnotes)

In this exchange, CHVs were overlooked in logistics, which was critical given CHVs found themselves posted outside of their areas:

[Yusuf] added that there was a vehicle at the dispensary which was used to transport the vaccines and the vaccinators but the CHVs were not included. ‘They have completely disrespected us; it's like our work is not important.’

CHVs are, on paper, the indispensable link between services and households where distrust of biomedical interventions run high. CHVs had a key role in garnering consent, including on the part of parents for children heading to school. Despite this, most CHVs summoned that day were expended, in a ‘denial of local capacity’ common to emergencies (Burgess, 2023, p. 68). In their agitation, CHVs heard and amplified rumours of outsiders undertaking mobilisation in their place, with variants that county government were to blame:

Our county is very corrupt; everyone brings in their own person to work but these positions are supposed to be for the CHVs.

Other variants suggested that this was outside county control. Regardless of such missteps, CHVs were not assigned to their home areas, restricting the work they could do:

Another female CHV joined us. She is attached to a different CU [health unit] but was asked to come work in this CU as someone else had taken *her* place. She was clearly not pleased and said that the community members would not accept any mobilization efforts from her, as they do not know her. She mentioned that in the past two days she had just been filling out the cards.

Another CHV found his name removed in a handwritten list of mobilisers, stepping in later as an ‘understudy’. When describing how he came to be involved:

He went on to mention that there were two lists of those recruited: one on the computer system and one handwritten. He found out that someone had replaced him in the handwritten list. He only ended up taking one of these CHV positions as [CHV] had been called to a different training. This made him very angry; the level of disorganization and unfair recruitment process made him speak with a lot of disgust.

As we return to in the discussion, dispensing with CHVs or their haphazard allocation outside of their area were cited as the biggest hindrances to the campaign.

### Community contention

The long-standing distrust of government fed into a ‘spectrum of contention’ on the part of communities, ranging from disquiet, evasion and refusal to get oneself or one’s children vaccinated. CHVs reported ‘accumulated contention’ in their communities, linking different campaigns and diseases, including COVID-19:

[When asked about YF response] They are becoming even more resistant. On COVID, when I assure them that the vaccine is safe, they refuse, citing concerns that it might cause infertility. (Interview)

There are those who refused [YF vaccine], and we had to use the nurse or PHO. Some would accept, others would refuse completely. They would say that these vaccines have been too many. They refused the COVID-19 vaccine and we had so many of those in the facility. (Interview)

Infertility fears, across diseases and campaigns, were recursive:

[When asked about YF rumours] They would equate it to HPV rumours that the Yellow Fever vaccine would bring infertility issues or some would say once you are vaccinated you will have malaria. (Interview)

Most of the time when there is a vaccination campaign, rumours spread that if you get vaccinated, you won’t be able to have children or it will cause illness. These rumours quickly enter people’s minds. (Interview)

Vaccination fears are global and side effects not unusual. Infertility fears here, however, link to specific histories of vaccinations in Africa, whereby campaigns were botched (Lowes & Montero, 2021) and rumours are strongly present in contexts of power asymmetries (Feldman-Savelsberg et al., 2000; White, 2008), particularly where, akin to Fanon’s idea of ‘omnipresent death’ (1965, p. 128), groups’ very survival has been put at risk (Fassin & Gomme, 2012; Kaler, 2009), discernible here:

[When asked if similarities between COVID and YF response] Community members were saying these vaccines were for family planning to stop people from giving birth and to make the population reduce or die at a faster rate. (Interview)

[When asked YF response] When you are mobilizing, some of the community members ask ‘what is that vaccine for? Do you want to finish us off?’ (Interview)

Local resistance to top-down vaccination campaigns remained a recurrent form of disavowal of colonial and postcolonial rule (Keller, 2006; Vaughan, 1992). We observed some evidence of withdrawal on the part of communities, via events at the school:

A total of 49 children were vaccinated although the class had 58 present. Some said they had been vaccinated the previous day in church while others had strict instructions from parents not to be given the jab. (Fieldnotes)

Relatedly, we observed more women than men availing of the vaccine:

I counted only 10 community members and they were women and children: no man visible to be vaccinated. This was our experience even in the other exercises we were part of; more women came forth to get the jab than men. (Fieldnotes).

Such campaigns leverage existing infrastructures centred on maternal and child health (Kaler, 2009), despite the fact that 88.7% of cases were male and mainly adult (Uwishema et al., 2022, p. 2).

Relatedly, a key form of outreach was the use of the school, which remains under national control, in order to build trust and further access. However, the use of the school overrode pushback from communities and foreclosed meaningful consent on the part of parents, leveraging a default ‘submission to authority’ (Feldman-Savelsberg et al., 2000, p. 165). Given that CHVs were not operating in their own area, this placed further onus on communities to access and process information, before getting word to the school to withdraw consent in advance of the team arriving (when this timetable was not known, even to teams). While we saw senior pupils refusing the vaccine, or sneak out of class, younger children could not. The stewarding of young pupils by teachers caused distress for some. We viewed the school’s discipline as key to the high numbers vaccinated:

The vaccinators were asking the children to line up. The class teacher was present and in control, long ruler in hand. The class was very quiet and you could hear a pin drop. The teacher was the one controlling the queue. (Fieldnotes)

In communities, including cases where children had missed vaccination at school, CHVs reported that local chiefs were key to mobilisation. Chiefs fall under the Ministry of Interior, via the County Commissioner’s office, with a long-established role in enforcing state decrees (Lonsdale & Berman, 1979; Wamagatta, 2009), including in health (Chaiken, 1998). They are well-trusted and practised in assembling the masses but also carry considerable power and influence, which can be difficult to refute or avoid.

However, this is not to overstate resistance and, as explored, there was a spectrum of contention. It was not necessarily that local people believed that YF was not *real*, which pertained to COVID and HPV, but questioned whether invasive vaccinations were justified*.* There was familiarity with YF, with local language names and often seen as a kind of malaria, yet the top-down drive to vaccinate caused consternation, particularly among older residents:

Those who refused would say that why should they inject, yet in the past they would never get injected, and yellow fever was in existence. It is like they were being forced. (Interview)

It is worth reiterating that this campaign took place at a time of pronounced stress on lives and livelihoods in Isiolo, whereby a fourth year of failed rains had led to drought and malnutrition, with almost two thirds of livestock reported to have been lost. Avoidance or refusal, therefore, may not be fuelled by ‘ignorance’ but rather a comment on the government’s ability to rally resources to intervene upon disease when deemed a threat, but not with regard to ecological crisis and suffering. As such, rumours are ‘not events misinterpreted and deformed, but rather events analyzed and commented upon’ (White, 2008, p. 58).

To return to Fanon, the use of national institutions with the ability to compel shows how the doctor, schoolteacher or chief remain the face of the otherwise distant state (1965, pp. 121–123). Understanding regimes of domination sheds light on a ‘complex of refusal on the part of the colonized’ (1965, p. 130). Dominant forces, however, are no longer visibly ‘foreign’, but viewed to reside within power centres to the south. While health is devolved, many staff were outsiders under national directives, so it was to *national* government that conspiracies were often directed, even with respect to the manifestly foreign COVID-19:

[Communities] viewed it as a business until coronavirus hit and people began dying. Only then did they start to believe. Before that, they thought it was just a government scheme. (Interview)

Precariously positioned between a historically punitive state and the masses are these volunteers, who face contention or even censure from their communities, as well as their *de facto* employers, leading to long-term insecurity and angst.

### Psychoexistential angst

CHWs undertake multiple roles, for multiple constituencies and have multiple paymasters. Their multi-positionality has been long-noted (e.g. Colvin & Swartz, 2015; Mlotshwa et al., 2015) but, rather than calling to capitalise on such to do *more* (cf. Haines et al., 2007), we underscore how long-term ambiguity becomes embodied and can cause harm (Maes et al., 2019). Further, community health work generates contention for CHVs on dual fronts: denigration from above and disavowal ‘from below’. In our first days of YF shadowing, we followed Yusuf and Zuleka who were experienced CHVs and often worked as a team. We had had recurrent prior interactions and had witnessed their ethic and skills that made them highly effective, yet their agency was constrained (Fanon, 1952), manifest in feelings of frustration and powerlessness that bubbled over daily. Yusuf reiterated during the campaign that they felt dehumanised by all agencies, stating: ‘they treat us like we are not human beings’ (fieldnotes). This was a recurring sentiment throughout the project, observing the regular refrain: ‘we are human beings too!’, manifest in a variety of ways.

First, during the campaign, long-standing resentments regarding the stature of CHVs’ work were exacerbated by the fact that response protocols had not provided guidance on essential activities to be performed specifically by CHVs. One flashpoint was the demotion of CHVs from mobilisers to scribes, writing names on lists or certificates:

They [vaccinators] look down on us; it's like our work is not important. Our work is to mobilise, not to write and record! (Fieldnotes)

While some reluctantly took up that role, others refused or subverted it, claiming to have no pen or – an ironic play on outsiders’ view of their lowly status – being unable to write. Yusuf felt disrespected by an incoming vaccinator who, in his view, wielded illegitimate authority:

I saw [out of earshot] that the first exchange between one vaccinator and Yusuf was not good. She gave him either some demeaning work or said it in a demeaning tone. He pushed back hard: that this was not his work, that he shouldn’t be spoken to in that way, that they were pivotal to the success of this process. […] He voiced that the vaccinator should know better than to address him with contempt as without them, no work would be carried out! ‘*We* are the voice in this community; they are not even known!’

Being left to wait for extended periods is part of CHV working life, which brings economic opportunity costs but also undermines their sense of worth, as borne out here:

Time kept on lapsing but no one from the vaccination team made an appearance. [Two CHVs] lamented how disrespectful they were for not communicating to them. They felt as if they were taken for granted. Two hours later, at around 12.30pm, the team appeared and acted as if nothing was wrong. […] The CHVs were visibly mad that [there had been] no form of communication, not even a phone call to alert them of the new developments. As they set up their station at the facility, [the CHVs] had lost their morale, mainly because the mobilised community members came and went because they did not find anyone there. They felt like their efforts were in vain.

Senior CHVs stated their recursive devaluing contributed to poor outbreak management:

When diseases break out, the response is a problem. You go [to communities] and come back, but there is disrespect. There’s also disrespect at the general hospital; people there don’t recognise our work, even though we do a lot of it in difficult conditions. So, the response is poor. (Interview)

However, the broader agitation cannot solely be attributed to poor outcomes. It requires unpacking perceived injustices regarding the costs of such work and the ‘psychosocial distress’ of its devaluing as dispensable and unskilled (Maes et al., 2019). At the nub of this was CHVs’ voluntary status and ambiguity around payment. For most, salaried payment would bring respect and clarity. For a few, voluntary service should bring esteem. Either way, the payment question went beyond material concerns to that of visibility and recognition (Dodworth & Mukungu, 2023; Mukungu et al., 2023), whether from above by government or below from communities:

We should be recognised by the community, not just treated with contempt as we currently are. We are treated that way because we are not paid for our work. (Interview)

People also look down on us because we are volunteers, they see us as insignificant. People think we have no jobs, so we volunteer to walk around visiting homes. We are seen as lowly, inferior people. (Interview)

You don't receive any payment, yet people still treat you with contempt. (Interview)

CHVs have thus been caught in a precarious middle, whereby over-professionalising this work risks alienating them from community members but under-professionalisation allows devaluing to take place. This devaluing allows CHVs not to be seen as workers, curtailing avenues of redress:

We make noise, and the officials have to follow up, meet with the higher-ups, and they end up being treated with contempt. There was a time [July 2021] we tried to demonstrate, and we were told, ‘CHVs, no one has employed you!’ (FGD)

When you say you’re a volunteer, it's recognised as a valuable role, but when it comes to discussing formal employment, it's a different matter entirely because this work is often devalued. You’re told, ‘but aren’t you just a volunteer?’ (Interview)

The erratic financial rewards, referred to as ‘tokens’, were experienced as demeaning rather than dignified, which speaks to a longer history of requisitioned public works in Kenya (Fall & Roberts, 2019).

Our main issue is with the tokens we receive. We are human beings too! (Interview)

Lastly, CHVs straddle multiple constituencies but also rationalities: one of the people but also enforcer of state-sanctioned interventions. Their embeddedness and trust with community members can allow them to engage with so-termed vaccine hesitancy productively (Shumba et al., 2024), yet this overlooks their own hesitancy, especially when working for external actors. Often, as in this campaign, the incentivised role of enforcer won out, embodied by CHVs’ stewarding of children to queues in their ‘assembling of the population’ (Fanon, 1965, p. 121). Our final excerpt returns to Fatuma, with whom our YF engagement started, who spent most of her time corralling queues in schools:

As we moved to the last and final grade for the day, Fatuma was busy ‘mobilising’ those students who had not been in school and missed the chance to get the jab. Some came willingly while others were dragged by their hands. (Fieldnotes)

## Discussion: coloniality, containment and care

We employed a Fanonian lens to probe the socio-political dynamics regarding a mass vaccination campaign in Isiolo. With YF an original ‘scare’ disease in international health, among ‘the most feared’ in the colonial era that looms large in today’s urbanizing world (Zhao et al., 2018), we see continuities with Fanon’s observations regarding top-heavy, externally-decreed responses against a backdrop of distrust. Indeed, Kenya’s north has been a ‘scare region’ historically: a repository of disease and conflict, whereby national government action is reactive and forceful. We benefit from recent work with respect to outsider-driven interventions to contain epidemics such as Ebola in West Africa (e.g. S. H. Ali & Rose, 2022; Farmer, 2021), and how community contention must be historicised, but the ‘outsider’ can come from within Kenya. We acknowledge limitations to our study, whereby we engaged only indirectly with community members and CHVs’ superiors, CHAs, who remained wary of our time with CHVs. Despite these limitations, we make the following contributions.

Regarding future campaigns, there are a number of ‘quick wins’ that can be derived from our findings. First, in respect to their displacement’, CHVs expressed they held little authority outside of their areas, eroding trust and denying local skills and capacity. The roles of CHVs in emergency response are not clearly stipulated within Kenya’s health policies (here, the action plan was compiled by the International Red Cross). They are folded into a homogenised ‘Community Health’, which houses differentials in power, positionality and interest. This lack of specificity led to CHVs being demoted to scribes, working with communities not known to them or being dispensed with entirely. Second, the leveraging of authoritative national institutions, *particularly* in the absence of CHVs working in their own areas, applies real-time pressure, undermines consent and reproduces the perception of force. Third, our findings build on previous studies whereby the vaccination architecture stems from that of maternal and child health, affirming long-standing fears regarding fertility and raising questions as to managing diseases that affect other groups. 88% of reported YF cases were (mainly adult) male but, while we do not make county-wide claims, the majority observed being vaccinated were women and children, with CHVs citing similar trends.

Fourth, the authors cannot offer an answer as to why Yellow Fever remains off the national childhood vaccination schedule, as compared to Ghana where vaccination rates are ∼88% (M. S. Ali & Mekonen, 2024). However, given our historicisation of this campaign and the literatures cited therein, containment and reactive responses to threat have proven intractable approaches to dealing with Kenya’s outreaches. In the case of YF, this is more expensive in the long-term and would be better served by prevention (Baba & Ikusemoran, 2017). Remarkably, according to one study on YF (that we do not believe pertains to Isiolo, where Community Health has been better embedded), the majority of mothers sampled in Kenya reported receiving no information from their CHV regarding any childhood vaccination (M. S. Ali & Mekonen, 2024, p. 3). The scope for an expanded Community Health approach appears considerable. To summarize these four points, the CHV’s place should be protected and capacitated to undertake the delicate, long-term mediations needed with communities to further public health, especially vaccinations.

More long-term, however, informed by Fanonian insights, we argue that the lack of any meaningful settlement between Kenya’s northeast and the postcolonial state remains a pervasive source of governmental distrust. Time-pressured campaigns within outbreak response follow colonial-like logics, whereby incomers disrupt community spaces via the same structures through which reproductive regulation take place, allowing the ‘conflation of contraception and vaccination’ (Kaler, 2009, p. 1717). State intervention has been sporadic and continues to oscillate between containment/neglect and emergency response. Relevant here, irrigation follies conceived by a distant state made the region more vulnerable to YF. The spectrum of responses to external intervention must be historicised in this way, explaining why vaccinations have been ‘one of the most resisted forms of state control’ (Feldman-Savelsberg et al., 2000, p. 171). Only when there is greater regional parity will such intrusions be trusted as locally pressing and relevant. Given the scale of environmental and humanitarian crises pastoralists face, there are few quick wins in this regard.

Last, we translate Fanon’s work to the everyday lives of CHVs, who must mediate between state-decreed interventions and their communities who remain wary of external interference, heightened in emergency response. CHVs assume dual positionalities: representative of the people, embedded in local cultures, as well as modernist and enforcer of state-sanctioned healthcare. Such contradictions were central to Fanon’s work on the fallout from continual internal confrontation (1952). CHVs clearly articulated their struggles ‘against sedimented, dehumanised constructions’ (Gordon, 2015, p. 50), via their lack of security, recognition and, ultimately, their *invisibility*. We witnessed anger and upset on the part of CHVs that pointed to larger, accumulated experiences than this campaign alone could explain, encapsulated by the refrain ‘we are humans too!’ heard regularly in meeting observations and interviews. We conclude that while CHVs’ interstitiality may well be an asset in ‘effectiveness’ terms, it subjects them to contention from above and below. Community Health is not just homogenised in Global Health; it is mythologised in ways that erase the contentions explored here and thus devalues their labours as frictionless, simply ‘going with the grain’.

As a concluding comment, we link our discussion to Fanon’s dialectic regarding liberation and the post-independence project. Fanon foresaw in national movements the impulse to exclude the masses on the basis of their pre-modernity, with elites passing ‘the same unfavourable judgement’ on the margins (1967, p. 87), borne out here in media representations and successive policymaking. National elites also bear responsibility to dismantle colonial ways of doing and seeing, which includes a prolonged and meaningful engagement with marginalised regions. As Mburu argued some decades ago: ‘If Kenyans … found the colonial health system unjust, they must also strive to create a system that is not disproportionately favourable to a few’ (1981, p. 527) and one that addresses environmental health and socio-economic deficiencies (1981, p. 525). The answers to just, appropriate and effective epidemic response lie in such principles but, more expansively, a collective care and a shared humanity that is still lacking within global governance today.

## Note

1 All fieldnotes drawn from the YF campaign period, 21–31 July 2022. Interviews span the full research period.
